# Chronic respiratory disease among the elderly in South Africa: any association with proximity to mine dumps?

**DOI:** 10.1186/s12940-015-0018-7

**Published:** 2015-04-03

**Authors:** Vusumuzi Nkosi, Janine Wichmann, Kuku Voyi

**Affiliations:** School of Health Systems and Public Health, Faculty of Health Sciences, University of Pretoria, P.O. Box 2034, Pretoria, 0001 South Africa

**Keywords:** Mine dumps, Chronic respiratory diseases, Elderly, South Africa

## Abstract

**Background:**

There is increasing evidence that environmental factors such as air pollution from mine dumps, increase the risk of chronic respiratory symptoms and diseases. The aim of this study was to investigate the association between proximity to mine dumps and prevalence of chronic respiratory disease in people aged 55 years and older.

**Methods:**

Elderly persons in communities 1-2 km (exposed) and 5 km (unexposed), from five pre-selected mine dumps in Gauteng and North West Province, in South Africa were included in a cross-sectional study. Structured interviews were conducted with 2397 elderly people, using a previously validated ATS-DLD-78 questionnaire from the British Medical Research Council.

**Results:**

Exposed elderly persons had a significantly higher prevalence of chronic respiratory symptoms and diseases than those who were unexposed., Results from the multiple logistic regression analysis indicated that living close to mine dumps was significantly associated with asthma (OR = 1.57; 95% CI: 1.20 – 2.05), chronic bronchitis (OR = 1.74; 95 CI: 1.25 – 2.39), chronic cough (OR = 2.02; 95% CI: 1.58 – 2.57), emphysema (OR = 1.75; 95% CI: 1.11 – 2.77), pneumonia (OR = 1.38; 95% CI: 1.07 – 1.77) and wheeze (OR = 2.01; 95% CI: 1.73 – 2.54). Residing in exposed communities, current smoking, ex-smoking, use of paraffin as main residential cooking/heating fuel and low level of education emerged as independent significant risk factors for chronic respiratory symptoms and diseases.

**Conclusion:**

This study suggests that there is a high level of chronic respiratory symptoms and diseases among elderly people in communities located near to mine dumps in South Africa.

**Electronic supplementary material:**

The online version of this article (doi:10.1186/s12940-015-0018-7) contains supplementary material, which is available to authorized users.

## Background

Chronic respiratory diseases are among the leading causes of death worldwide [1]. A recent review indicated that developing countries are experiencing an increase in the prevalence of respiratory diseases [2] and projected trends of severity and frequency are likely to pose a public health challenge [3]. Studies have shown that both indoor and outdoor air pollution are the main risk factors for the burden of respiratory diseases [4,5] and elderly people are mostly affected [6] as a result of normal and pathological ageing [7]. A higher burden of respiratory diseases among the elderly could be of concern to South Africa’s rapidly increasing population aged 60 years and above, currently the second largest in sub-Saharan Africa [8].

In developing countries where health risks of air pollution may be underappreciated and effective air pollution abatement techniques are lacking, people are continually exposed to concentrations that can have negative health effects in both the short and long term. Various risk factors have been associated with chronic respiratory diseases, including gender [9], socio-economic status [10], tobacco smoking habits [11], occupational environment [12] and polluting fuel used for residential cooking/heating [13]. Studies conducted in South Africa on the prevalence of respiratory diseases have been in industrialized urban areas [14,15].

Mine dump facilities are the main source of airborne particulate matter pollution, the dust is blown into the surrounding communities and can potentially have adverse health effects on human health and ecology [16,17]. Communities located close to mine dumps are of lower socio-economic status, often children and the elderly. These communities consist of historically disenfranchised ethnic groups living in government-funded houses, informal settlements and retired homes [18]. Epidemiological studies have shown that residing near mines is a major risk for exposure to particulate matter and metals such as cadmium, lead, silica, manganese, lead and arsenic [19-21]. Exposure to mine dump dust that is that is rich in silica has been linked to the development of chronic bronchitis, emphysema and airflow obstruction [22]. Settle-able dust has a negative effect on visibility, when it forms dust plumes while its deposition on fabrics, buildings, vehicles and water tanks constitutes a nuisance [23]. The ongoing reclamation of mine dumps for gold recovery observed during the survey, is worsening dust pollution with further deterioration of ambient air quality in the study populations. Many epidemiological studies have linked the effects of ambient air pollution with respiratory diseases [24,25]. Elderly people are potentially highly vulnerable to the effect of ambient air pollution, due to normal and pathological aging [26].

No studies have investigated whether exposure to mine dust or living in close proximity to mine dumps poses an increased risk for respiratory diseases among elderly people or possible effect modifications between various air pollution sources, including mine dust.

This study is part of the bigger project initiated by Mine Health Safety Council of South Africa (MHSC) around communities located near mine dumps in Gauteng and North West provinces. It is, to the best of our knowledge, the first study that has investigated the association between potential risk factors and chronic respiratory diseases among elderly people staying in communities situated near mine dumps in South Africa. The aim of the study was to investigate whether the prevalence of chronic respiratory symptoms and diseases among the elderly community were associated with proximity to mine dumps. Effect modification between proximity to mine dumps and other air pollution sources was also investigated, for instance the type of fuel use for residential cooking/heating, tobacco smoking and history of occupational exposure to dust or chemical fumes.

## Methods

### Study area and demographics

**Figure 1:**
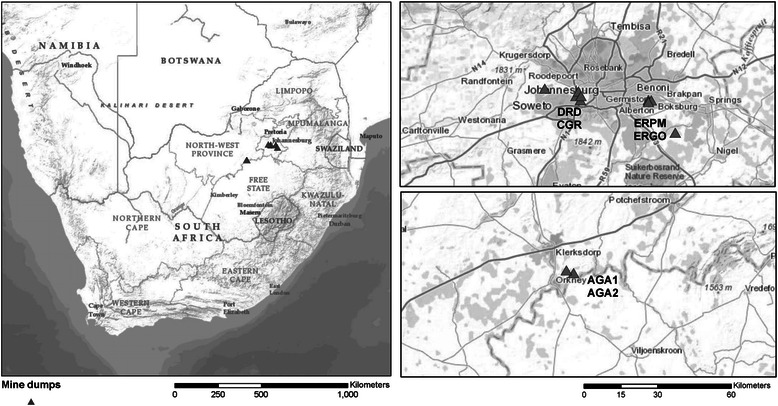
**Location of mine dumps tailings in South Africa.**

Communities living 1-2 km (exposed) and 5 km (unexposed) from five pre-selected mine dumps in Gauteng and North West Provinces of South Africa were studied during November and December in 2012. Table 1 lists the selected communities and Figure 1 shows a map of the study area. The socio-economic and demographic profile of exposed and unexposed communities was similar.

**Table 1:** **Eleven communities selected in the study located in Gauteng and North West provinces, South Africa during November-December 2012**

Mine dump facility	Province	Exposed communities ^ a ^	Unexposed communities ^ b ^
Durban Roodepoort Deep (DRD)	Gauteng	Braamfischerville	Dobsonville
Crown Gold Recoveries (CGR)	Gauteng	Diepkloof, Riverlea, and Noordgesig	Orlando East
East Rand Proprietary Mines (ERPM)	Gauteng	Reiger Park	Windmill Park
Ergo	Gauteng	Geluksdal	Windmill Park
Anglo Gold Ashanti (AGA)	North West	Stilfontein	Jouberton

^a^1-2 km from mine dumps.

^b^5 km or more from mine dumps.

### Study design and sample selection

A cross-sectional epidemiological study design was applied. Face-to-face interviews were conducted using a previously validated ATS-DLD-78 questionnaire from the British Medical Research Council (BMRC) [27]. The study focused on elderly people (55 years old and above) who had been residing for a period of five years or more, in the study communities. A knock on the door approach was used to recruit study participants. The interviews were mainly in English and were translated into the local language if the respondent did not understand the questions.

Streets were randomly selected in each community. Four to five houses were then randomly selected in each street in a radial fashion. The sample size of each community was calculated using Epi Info version 7, with a total sample size of 3069. The population sizes were based on the 2001 census from Statistics South Africa because the results for census 2011 were not released when the study commenced. Twenty-two locally trained fieldworkers were employed, two per community listed in Table 1. Each fieldworker received thorough training in conducting the interviews using the respiratory-health questionnaire, before the start of the survey.

### Exclusion criteria

If a selected household had no elderly people or no one at home during the visit, or were unwilling to participate, the fieldworker proceeded to the next household.

### Quality control

To maintain the quality of the interviews, fieldworkers randomly selected 10% of homes and re-administered the same questionnaire to the same previously interviewed participants to verify their responses. This was performed 15 to 20 days after the first interview. Ten percent deviations within the interviews were deemed unacceptable.

### Health outcomes

Having asthma, chronic bronchitis, chronic cough, emphysema, pneumonia and wheeze were classified on the basis of positive answers to the following questions:

Asthma: “Was the asthma confirmed by the doctor?”

Chronic bronchitis: “Was the chronic bronchitis confirmed by the doctor?”

Chronic cough: “Do you cough most days for 3 consecutive months or more during the year?”

Emphysema: “Was the emphysema confirmed by the doctor?”

Pneumonia: “Was the pneumonia confirmed by the doctor?”

Wheeze: “Does your chest ever sound whistling most days or nights?”

### Main exposure factor

The main exposure factor of interest in this study was based on the distance between the study population and a mine dump (Table 1).

### Confounders

Potential confounding variables included other air pollution sources: active smoking by participants (yes/no), main type of residential cooking/heating fuel (electricity, gas, paraffin, wood/coal). Additional confounders considered were: age (years), level of education (no schooling, primary, secondary, tertiary), sex (female/male), occupational exposure history to dust/chemical fumes (yes/no).

### Statistical analyses

Two technicians entered the collected questionnaire data into a database set up in Epi Info version 3.5.3. Data were analyzed using Stata version 12. Prevalence of the health outcome; the proportion of air pollution sources under investigation; and confounding variables, were calculated by dividing the number of participants who responded affirmatively by the number of questionnaires completed. Therefore each question had a different sample size. Observations marked as “do not know”, “not stated”, or “other responses” were set as missing, but were included in the descriptive analyses. Only two explanatory variables such as age and main residential heating and cooking fuel type had missing observations. Responses to the number cigarettes smoked per day were very low and not included in the analysis. A chi square test was applied, to determine the relationship between community (exposed/unexposed) and confounding variables. Crude and adjusted odds ratios (ORs) and 95% confidence intervals (CI) were calculated using univariate and multiple logistic regression analysis (LRA) to estimate the likelihood of having asthma, chronic bronchitis, chronic cough, emphysema, pneumonia and wheeze. Missing values were automatically excluded in each LRA model; therefore each multiple LRA model had a different sample size. To obtain adjusted ORs for the effect of “community (exposed/unexposed)” on the outcomes were placed in an initial LRA model. This was followed by the addition of a potential confounder in a stepwise manner starting with the most statistical significant from the univariate analysis. Each time a new potential confounder was added to the model if the effect estimate between the exposure of interest and respiratory outcome already in the models changed by more than 5%, the additional variable was retained in the final multiple LRA otherwise the variable was removed and a different one was added [28]. The most parsimonious multiple LRA models were reported, i.e. those with variables having a p-value < 0.05 [29]. Community (exposed/unexposed) was considered as the main exposure factor and therefore was included in all models for each outcome of interest regardless of whether it was statistically significant in the univariate analyses.

Effect modification between community (exposed/unexposed) and other air pollution source variables, such as smoking habits, occupational exposure history to dust/chemical fumes, and residential cooking/heating fuel type, was investigated by including a multiplicative term in the model.

### Ethics approval

Ethical approval (number: 235/2011) was obtained from the Research Ethics Committee, Faculty of Health Sciences, University of Pretoria. A verbal and written consent was obtained before commencement of the interviews.

## Results

Only completed questionnaires (2397) were used for data analysis. This included 1499 (63%) study participants from exposed and 898 (37%) from unexposed communities (Table 2). The number of females in the study was slightly higher than males. Most of the study participants were in the age group 55 to 59 years. Overall, the majority of participants from both study communities had obtained secondary level education. The proportion of current smokers and those with a history of occupational exposure history to dust/chemical fumes, in exposed communities, was double that of the unexposed. A majority of participants from both exposed and unexposed communities reported electricity as the main source of residential fuel for heating/cooking.

**Table 2:** **Demographic characteristics and air pollution variables by type of community in Gauteng and North West provinces, South Africa during November-December 2012**

	Community		p-value ^ c ^
	Exposed ^ a ^ (n = 1499)	Unexposed ^ b ^ (n = 898)	
***Sex***			
Female	774 (51.6)	472 (52.3)	0.66
Male	725 (48.4)	426 (47.4)	
***Age (in years)***			
55-59	500 (33.4)	225 (25.1)	<0.001
60-64	405 (27.0)	221 (24.6)	
65-69	228 (15.2)	125 (13.9)	
70-84	309 (20.6)	278 (31.0)	
≥85	48 (3.2)	29 (3.2)	
Missing	9 (0.6)	20 (2.2)	
***Population group***			< 0.001
Black	1006 (41.9)	695 (29.0)	
Coloured	493 (20.6)	203 (8.5)	
***Level of education***			
No schooling	262 (17.5)	271 (30.2)	<0.001
Primary	479 (32.0)	287 (32.0)	
Secondary	691 (46.1)	332 (37.0)	
Tertiary	67 (4.5)	8 (0.8)	
***Smoking habits***			
Non-smoker	888 (59.2)	598 (66.6)	<0.001
Ex-smoker	234 (15.6)	187 (20.8)	
Current smoker	377 (25.2)	113 (12.6)	
***Occupational exposure history to dust/chemical fumes***			
Yes	637 (42.5)	149 (16.6)	<0.001
No	862 (57.5)	749 (83.4)	
***Main residential heating/cooking fuel type***			
Electricity	1422 (94.9)	783 (87.2)	<0.001
Gas	31 (2.1)	67 (7.5)	
Paraffin	25 (1.7)	6 (0.7)	
Open fires	1 (0.07)	13 (1.5)	
Missing	20 (1.3)	29 (3.2)	

Figures in parentheses are percentages.

^a^Exposed: communities located 1–2 km from mine dumps.

^b^Unexposed: communities located 5 km or more from mine dumps.

^c^p-values of the Chi-square test.

**Figure 2:**
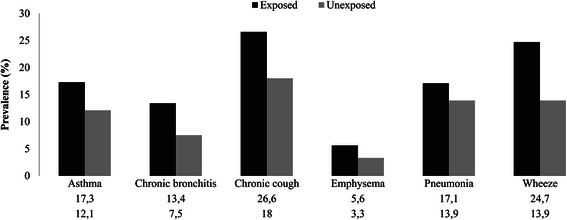
**Prevalence of chronic respiratory symptoms and diseases stratified by type of community located 1-2 km and ≥5 km from mine dumps in Gauteng and North West provinces, South Africa during November-December 2012.**

The prevalence of asthma (17.3%), chronic bronchitis (13.4%), chronic cough (26.6%), emphysema (5.6%), pneumonia (17.1%) and wheeze (24.7%), in the exposed communities was higher than that of the unexposed communities, where the proportions were 12.1%, 7.5%, 18%, 3.3%, 13.9% and 19.3%, respectively (Figure 2).

The prevalence of asthma, chronic bronchitis, chronic cough, emphysema, pneumonia, and wheeze per each risk factor considered in this study are shown in Additional file 1: Table S1. Crude and adjusted ORs for all risk factors except the main exposure factor are shown in Additional file 2: Tables S2 and Additional file 3: Table S3.

Results from the multiple LRA (Table 3) indicated that living in the exposed communities was significantly associated with asthma (OR = 1.57; 95% CI: 1.20 – 2.05), chronic bronchitis (OR = 1.74; 95% CI: 1.25 – 2.39), chronic cough (OR = 2.02; 95% CI: 1.58 – 2.57), emphysema (OR = 1.75; 95% CI: 1.11 – 2.77), pneumonia (OR = 1.38; 95% CI: 1.07 – 1.77) and wheeze (OR = 2.01; 95% CI: 1.73 – 2.54). Sex was not associated with any of the health outcomes considered in this study. The study participants who were in the age group between 70–84 years were at an increased likelihood of having chronic bronchitis (67%), emphysema (81%) and pneumonia (36%). Coloured participants were less like to experience cough (OR = 0.55; 95% CI: 0.42 – 0.71) and wheeze (OR = 0.54; 95% CI: 0.44 – 0.66) as compared to black. Participants with primary level as their highest education were 50% more likely to have asthma. Having secondary education was associated with chronic bronchitis (OR = 1.45; 95% CI: 1.01 – 2.22) and wheeze (OR = 1.54; 95% CI: 1.54 – 1.98). Current and ex-smoking significantly increased the likelihood of having chronic cough, wheeze, asthma, emphysema, pneumonia, and were not associated with chronic bronchitis respectively. Occupational exposure history to dust/chemical fumes was significantly associated with chronic bronchitis (OR = 1.43; 95% CI: 1.07 – 1.91). Using polluting fuels, such as paraffin or gas for residential cooking/heating had significant detrimental association with chronic cough (OR = 2.03; 95% CI: 1.13 – 4.78) and pneumonia (OR = 2.40; 95% CI: 1.11 – 5.17) (Additional file 3: Table S3).

**Table 3:** **Univariate and multivariate analyses of chronic respiratory symptoms/diseases and diseases in all 11-study communities located 1-2 km and ≥5 km** ^*^ **from mine dumps in Gauteng and North West provinces, South Africa during November-December 2012**

*Chronic respiratory symptoms/diseases*	*Crude OR*	*(95% CI)*	*p-value*	*Adjusted OR*	*(95% CI)*	*p-value*
Asthma^a^	1.49	(1.17 – 1.90)	0.001	1.57	(1.20 – 2.05)	0.001
Chronic bronchitis^b^	1.92	(1.44 – 2.57)	<0.001	1.74	(1.26 – 2.39)	0.001
Chronic cough^c^	1.77	(1.43 – 2.20)	<0.001	2.02	(1.58 – 2.57)	<0.001
Emphysema^d^	1.72	(1.12 – 2.63)	0.013	1.75	(1.11 – 2.77)	0.016
Pneumonia^e^	1.25	(1.01 – 1.58)	0.018	1.38	(1.07 – 1.77)	0.014
Wheeze^f^	1.96	(1.65 – 2.32)	<0.001	2.01	(1.73 – 2.54)	<0.001

*Communities located ≥5 km (unexposed) from mine dumps used as reference category.

^a-f:^Models adjusted for sex, age, population group, smoking habits, occupational exposure history to dust/chemical fumes and main residential heating/cooking fuel type.

No significant effect modification between community type (exposed/unexposed) and other air pollution source variables was observed (results not shown).

## Discussion

This is the first study that investigated the prevalence and risk factors associated with chronic respiratory symptoms and diseases and among elderly people in communities exposed to mine dumps in South Africa. The results of this study suggest that there is a high prevalence of asthma, chronic bronchitis, chronic cough, emphysema, pneumonia and wheeze in the seven exposed communities. Residing in exposed communities, smoking habits, use of paraffin for residential cooking/heating, and having a low level of education emerged as significant risk factors for chronic respiratory symptoms and diseases.

The risk of exposure to particulate matter from mine dumps is well documented by international research studies [30-32]. An exposure assessment study done in one of the mine dumps in this study showed that the ambient concentration of particulate matter with an aerodynamic diameter less than 10 μm (PM_10_) exceeded by far the 24-hour limit set by the South African Department of Environmental Affairs (180 μg.m^−3^) [16,33]. Residential developments in some communities are a stone throw from the mine dump, resulting in elevated exposure to particulate matter [34]. Therefore, respiratory diseases and symptoms could be aggravated or originated as a result of exposure to dust emanating from mine dumps.

No significant differences were observed between the prevalence of chronic respiratory symptoms and diseases in males and females, and sex was not associated with any of the health outcomes considered in this study. Previous studies have reported that males were at increased risk for respiratory diseases [35,36]. This difference may result from differential occupational exposure rates and smoking between males and females.

Aging is normally associated with an increased risk of respiratory symptoms and diseases [36,37], this might be attributed to anatomical, physiological and immunological changes that occur in the respiratory system during aging [38]. In this study increase in age was not significantly associated with having chronic respiratory symptoms and diseases, survivor effect could be a possible explanation for these observations. A research study conducted in South Africa showed being coloured was associated with the presence chronic lung diseases among the elderly people [39]. However, in this study a significant protective effect was observed. Primary and secondary education levels were significant risk factors for respiratory symptoms and diseases. Lower education levels are known to be linked to low socio-economic status and have been identified as a risk factor for respiratory symptoms and diseases [40]. A national household survey conducted in South Africa reported higher education level as a protective factor for respiratory diseases [41]. The findings of this study support this association.

Association of smoking habits with respiratory diseases and symptoms is not novel [42-44]. Current and ex-smoking was significantly associated with chronic cough, wheeze, asthma, emphysema and pneumonia. Ex-smokers were at a higher risk than current smokers of having the latter. Being diagnosed as having chronic respiratory symptoms and disease might be the reason why respondents stopped smoking. Another possibility might have been advice from doctors, as it has been shown that physician’s advice could contribute to smoking cessation [45].

The respiratory system is susceptible to harm from occupational exposures due to direct contact with the ambient environment, and inhalation of possible toxic substances [46]. Occupational exposure history to dust/chemicals was not associated with respiratory diseases or symptoms. The findings of this study are in contrast to those of other research studies in this respect [47-49]. Domestic use of paraffin or gas appliances has been associated with respiratory symptoms and diseases in children, less consistently with adults and elderly people [50,51]. In this study domestic use of paraffin and gas was associated with an increased risk of respiratory symptoms and diseases, possibly caused by oxides of nitrogen or carbon monoxide generated when gas or paraffin is burned [51].

This study has some limitations inherent to cross-sectional epidemiological designs. Firstly, the study cannot provide any evidence of causality. Secondly, no quantitative air pollution exposure assessment was conducted. Thirdly, we relied on doctor diagnosed respiratory diseases, which, although specific, can cause an underestimation of disease prevalence. Therefore it is assumed that several individuals with respiratory diseases were missed due to restrictive definitions employed in this study, as a result of poor access to health care facilities associated with black poor communities [52]. The possibility of estimating falsely low prevalence figures cannot be ignored. However, the observed high prevalence of respiratory diseases in exposed communities cannot only be attributed to a strict definition used, but to a complex interaction of social, economic, and behavioral factors such as air pollution, under-nutrition, poor access to healthcare, or life-style behaviors [53,54]. Fourthly, the interviewer error might have occurred in the translation of the questions to the local language during the interview of some study participants who did not understand English. Fifthly, unwillingness of the respondents to provide honest answers or giving socially desirable responses should be taken into account in the interpretation of the results. Sixthly, no lung function tests and/or spirometry, were conducted during the study. Lastly, the differential participation rate between exposed and unexposed communities is of concern and may well have introduced response bias, which is likely to overestimate the prevalence estimates derived from our cross-sectional study and also bias the association in either direction.

An advantage of this study is that 10% of the study participants were interviewed twice, with 96% repeatability observed.

## Conclusion

The study findings suggest that there is a high prevalence chronic respiratory symptoms and diseases among the elderly in communities located near mine dumps. The significant risk factors are proximity to mine dump, smoking habits, low level of education and domestic use of gas or paraffin.

## Additional files

Additional file 1: Table S1. Prevalence of chronic respiratory symptoms and diseases in relation to independent variables in all 11-study communities located 1-2 km and ≥5 km from mine dumps in Gauteng and North West provinces, South Africa during November-December 2012. Additional file 2: Table S2. Crude odds ratios with 95% confidence intervals of chronic respiratory symptoms and diseases in all 11-study communities located 1-2 km and ≥ 5km from mine dumps in Gauteng and North West provinces, South Africa during November-December 2012. Additional file 3: Table S3. Adjusted odds ratios with 95% confidence intervals of chronic respiratory symptoms and diseases in all 11-study communities located 1-2 km and ≥5 km from mine dumps in Gauteng and North West provinces, South Africa during November-December 2012.

## Abbreviations

CI: Confidence intervals
MLRA: Multilevel logistic regression analysis
AGA: Anglo gold ashanti
CGR: Crown gold recoveries
DRD: Durban roodepoort deep
ERPM: East rand proprietary mines
SSA: Statistics South Africa
NRF – DAAD: National research fund – deutscher akademischer austausch dienst
