# Supplementary material for: Chronic respiratory disease among the elderly in South Africa: any association with proximity to mine dumps?
Source: Environ Health. 2015 Apr 3;14:33. doi: 10.1186/s12940-015-0018-7 (PMC4406017; doi:10.1186/s12940-015-0018-7)
Supplement: Additional file 1: Table S1. — Prevalence of chronic respiratory symptoms and diseases in relation to independent variables in all 11-study communities located 1-2 km and ≥5 km from mine dumps in Gauteng and North West provinces, South Africa during November-December 2012. [file 12940_2015_18_MOESM1_ESM.docx]

**Table S1: Prevalence of chronic respiratory symptoms and diseases in relation to independent variables in all 11-study communities located 1-2km and ≥5km from mine dumps in Gauteng and North West provinces, South Africa during November-December 2012**

|  | **Chronic respiratory symptoms and diseases, *n (%)*** | | | | | |
| --- | --- | --- | --- | --- | --- | --- |
|  | **Asthma** | **Chronic bronchitis** | **Chronic cough** | **Emphysema** | **Pneumonia** | **Wheeze** |
| **Sex** |  |  |  |  |  |  |
| Male | 175 (7.3) | 125 (5.2) | 292 (12.2) | 56 (2.3) | 125 (5.2) | 255 (10.6) |
| Female | 194 (8.1) | 143 (6.0) | 268 (11.2) | 58 (2.4) | 143 (6.0) | 288 (12.0) |
| ***Age (in years)*** |  |  |  |  |  |  |
| 55 – 59 | 10.1 (4.2) | 75 (3.1) | 153 (6.4) | 27 (1.1) | 102 (4.3) | 149 (6.2) |
| 60 – 64 | 98 (4.1) | 58 (2.4) | 141 (5.9) | 25 (1.0) | 104 (4.3) | 147 (6.1) |
| 65 – 69 | 61 (2.5) | 36 (1.5) | 97 (4.1) | 24 (1.0) | 56 (2.3) | 74 (3.1) |
| 70 – 84 | 97 (4.1) | 86 (3.6) | 148 (6.2) | 37 (1.5) | 106 (4.4) | 148 (6.2) |
| 85 and above | 9 (0.4) | 12 (0.5) | 18 (0.8) | 1 (0.04) | 12 (0.5) | 18 (0.8) |
| ***Level of education*** |  |  |  |  |  |  |
| No schooling | 7.5 (3.1) | 44 (1.8) | 110 (4.6) | 26 (1.1) | 80 (3.3) | 81 (3.4) |
| Primary | 153 (6.4) | 82 (3.4) | 220 (9.2) | 44 (1.8) | 131 (5.5) | 155 (6.5) |
| Secondary | 292 (12.2) | 138 (5.8) | 219 (9.1) | 43 (1.8) | 159 (6.6) | 219 (9.1) |
| Tertiary | 15 (0.6) | 6 (0.3) | 11 (0.5) | 1 (00.4) | 12 (0.5) | 11 (0.5) |
| ***Smoking habits*** |  |  |  |  |  |  |
| Non-smoker | 211 (8.8) | 165 (6.9) | 299 (12.5) | 230 (9.6) | 230 (9.6) | 293 (12.2) |
| Ex-smoker | 80 (3.3) | 45 (1.9) | 127 (5.3) | 86 (3.6) | 87 (3.6) | 108 (4.5) |
| Current smoker | 78 (3.3) | 58 (2.4) | 134 (5.6) | 66 (2.8) | 58 (2.4) | 142 (5.9) |
| ***Occupational exposure history to dust/chemical fumes*** |  |  |  |  |  |  |
| No | 229 (9.6) | 158 (6.6) | 356 (14.9) | 71 (3.0) | 158 (6.6) | 344 (14.4) |
| Yes | 140 (5.8) | 110 (4.6) | 204 (8.5) | 43 (1.8) | 110 (4.6) | 199 (8.3) |
| ***Main residential heating/cooking fuel type*** |  |  |  |  |  |  |
| Electricity | 335 (14.0) | 242 (10.1) | 522 (21.8) | 108 (4.5) | 344 (14.4) | 509 (21.2) |
| Gas | 20 (0.8) | 13 (0.5) | 20 (0.8) | 3 (0.1) | 19 (0.8) | 17 (0.7) |
| Paraffin | 8 (0.3) | 4 (0.2) | 13 (0.1) | 2 (0.08) | 10 (0.4) | 10 (0.4) |
| Open fires | 2 (0.08) | 1 (0.04) | 2 (0.08) | 0 (0.0) | 4 (0.2) | 1 (0.04) |
