# Supplementary material for: Chronic respiratory disease among the elderly in South Africa: any association with proximity to mine dumps?
Source: Environ Health. 2015 Apr 3;14:33. doi: 10.1186/s12940-015-0018-7 (PMC4406017; doi:10.1186/s12940-015-0018-7)
Supplement: Additional file 2: Table S2. — Crude odds ratios with 95% confidence intervals of chronic respiratory symptoms and diseases in all 11-study communities located 1-2 km and ≥ 5km from mine dumps in Gauteng and North West provinces, South Africa during November-December 2012. [file 12940_2015_18_MOESM2_ESM.docx]

**Tables S2: Crude odds ratios with 95 % confidence intervals of chronic respiratory symptoms and diseases in all 11-study communities located 1-2km and ≥5km from mine dumps in Gauteng and North West provinces, South Africa during November-December 2012**

|  | **Asthma^a^** | **Chronic bronchitis^b^** | **Chronic cough^c^** | **Emphysema^d^** | **Pneumonia^e^** | **Wheeze^f^** |
| --- | --- | --- | --- | --- | --- | --- |
| ***Sex*** |  |  |  |  |  |  |
| Male | 1 | 1 | 1 | 1 | 1 | 1 |
| Female | 1.02 (0.81 – 1.27) | 1.06 (0.82 – 1.37) | 0.78 (0.63 – 0.95) | 0.95 (0.66 – 1.39) | 0.93 (0.744 – 1.15) | 0.86 (0.73 – 1.01) |
| ***Age (in years)*** |  |  |  |  |  |  |
| 55 – 59 | 1 | 1 | 1 | 1 | 1 | 1 |
| 60 – 64 | 1.14 (0.85 – 1.55) | 0.88 (0.62 – 1.27) | 0.98 (0.75 – 1.29) | 1.08 (0.62 – 1.87) | 1.22 (0.91 – 1.65) | 0.97 (0.78 – 1.21) |
| 65 – 69 | 1.29 (0.92 – 1.83) | 0.98 (0.65 – 1.50) | 1.34 (0.98 – 1.82) | 1.88 (1.07 – 3.31) | 1.16 (0.81 – 1.65) | 0.99 (0.76 – 1.27) |
| 70 – 84 | 1.23 (0.91 – 1.67) | 1.48 (1.07 – 2.07) | 1.30 (0.90 – 1.55) | 1.74 (1.05 – 2.89) | 1.35 (1.01 – 1.82) | 0.86 (0.69 – 1.07) |
| 85 and above | 0.82 (0.39 – 1.71) | 1.60 (0.82 – 3.09) | 1.19 (0.66 – 2.14) | - | 1.12 (0.59 – 2.15) | 0.74 (0.46 – 1.21) |
| ***Population group*** |  |  |  |  |  |  |
| Black | 1 | 1 | 1 | 1 | 1 | 1 |
| Coloured | 0.74 (0.57 – 0.96) | 1.74 (1.34 – 2.26) | 0.61 (0.49 – 0.77) | 0.78 (0.51 – 1.21) | 1.15 (0.90 – 1.44) | 0.70 (0.55 – 0.84) |
| ***Level of education*** | § |  |  |  |  |  |
| No schooling | 1 | 1 | 1 | 1 | 1 | 1 |
| Primary | 1.46 (1.08 – 1.97) | 1.30 (0.88 – 1.91) | 1.36 (1.02 – 1.80) | 1.19 (0.72 – 1.96) | 1.12 (0.83 – 1.52) | 1.43 (1.14 – 1.8) |
| Secondary | 0.84 (0.62 – 1.15) | 1.73 (1.21 – 2.48) | 0.87 (0.81 – 1.36) | 0.86 (0.52 – 1.41) | 0.99 (0.74 – 1.34) | 1.27 (1.02 – 1.57) |
| Tertiary | 0.99 (0.50 – 1.97) | 0.97 (0.40 – 2.35) | 0.60 (0.30 – 1.20) | 0.26 (0.04 – 1.97) | 1.03 (0.53 – 1.99) | 1.41 (0.87 – 2.30) |
| ***Smoking habits*** |  |  |  |  |  |  |
| Non-smoker | 1 | 1 | 1 | 1 | 1 | 1 |
| Ex-smoker | 1.39 (1.05 – 1.84) | 0.96 (0.68 – 1.36) | 1.47 (1.14 – 1.89) | 2.07 (1.31 – 3.29) | 1.38 (1.05 – 1.82) | 0.84 (0.68 – 1.05) |
| Current smoke | 1.12 (0.85 – 1.49) | 1.07 (0.78 – 1.48) | 1.43 (1.16 – 1.83) | 1.83 (1.16 – 2.88) | 0.85 (0.63 – 1.14) | 1.34 (1.11 – 1.67) |
| ***Occupational exposure history to dust/chemical fumes*** |  |  |  |  |  |  |
| No | 1 | 1 | 1 | 1 | 1 | 1 |
| Yes | 1.39 (1.05 – 1.84) | 1.5 (1.15 – 1.94) | 1.2 (1.01 – 1.51) | 1.3 (0.85 – 1.85) | 0.8 (0.67 – 1.07) | 1.3 (1.05 – 1.58) |
| ***Main residential heating/cooking fuel type*** |  |  |  |  |  |  |
| Electricity | 1 | 1 | 1 | 1 | 1 | 1 |
| Gas | 1.4 (0.85 – 2.36) | 1.2 (0.68 – 2.26) | 0.8 (0.50 – 1.36) | 0.6 (0.19 – 1.97) | 1.3 (0.77 – 2.16) | 0.68 (0.40 – 1.15) |
| Paraffin | 2.0 (0.89 – 4.57) | 1.2 (0.42 – 3.46) | 2.3 (1.13 – 4.78) | 1.3 (0.32 - 5.68) | 2.6 (1.19 – 5.49) | 1.6 (0.71 – 3.24) |
| Open fires | 0.9 (0.21 – 4.15) | 0.6 (0.81 – 4.79) | 0.9 (0.24 – 3.16) | - | 2.2 (0.67 – 6.89) | 0.22 (0.03 - 1.89) |

1:Reference category
