# Supplementary material for: Chronic respiratory disease among the elderly in South Africa: any association with proximity to mine dumps?
Source: Environ Health. 2015 Apr 3;14:33. doi: 10.1186/s12940-015-0018-7 (PMC4406017; doi:10.1186/s12940-015-0018-7)
Supplement: Additional file 3: Table S3. — Adjusted odds ratios with 95% confidence intervals of chronic respiratory symptoms and diseases in all 11-study communities located 1-2 km and ≥5 km from mine dumps in Gauteng and North West provinces, South Africa during November-December 2012. [file 12940_2015_18_MOESM3_ESM.docx]

**Tables S3: Adjusted odds ratios with 95 % confidence intervals of chronic respiratory symptoms and diseases in all 11-study communities located 1-2km and ≥5km from mine dumps in Gauteng and North West provinces, South Africa during November-December 2012**

|  | **Asthma^a^** | **Chronic bronchitis^b^** | **Chronic cough^c^** | **Emphysema^d^** | **Pneumonia^e^** | **Wheeze^f^** |
| --- | --- | --- | --- | --- | --- | --- |
| ***Sex*** |  |  |  |  |  |  |
| Male | 1 | 1 | 1 | 1 | 1 | 1 |
| Female | 1.13 (0.88 – 1.42) | 1.08 (0.82 – 1.41) | 0.87 (0.70 – 1.08) | 1.07 (0.72 – 1.61) | 0.87 (0.69 – 1.10) | 0.97 (0.82 – 1.16) |
| ***Age (in years)*** |  |  |  |  |  |  |
| 55 – 59 | 1 | 1 | 1 | 1 | 1 | 1 |
| 60 – 64 | 1.14 (0.83 – 1.55) | 0.93 (0.82 – 1.27) | 0.95 (0.72 – 1.25) | 1.04 (0.60 – 1.82) | 1.23 (0.91 – 1.66) | 1.04 (0.83 – 1.30) |
| 65 **–** 69 | 1.28 (0.89 – 1.82) | 1.06 (0.69 – 1.62) | 1.33 (0.97 – 1.83) | 1.78 (1.07 – 3.16)^*^ | 1.15 (0.81 – 1.65) | 1.05 (0.84 – 1.37) |
| 70 – 84 | 1.30 (0.95 – 1.78) | 1.67 (1.17 – 2.36)^*^ | 1.40 (1.05 – 1.86)^*^ | 1.81 (1.08 – 3.08)^*^ | 1.36 (1.01 – 1.85)^*^ | 1.08 (0.85 – 1.34) |
| 85 and above | 0.73 (0.33 – 1.59) | 1.89 (0.96 – 3.77) | 1.38 (0.74 – 2.54) | - | 1.07 (0.54 – 1.51) | 0.79 (0.47 – 1.33) |
| ***Population group*** |  |  |  |  |  |  |
| Black | 1 | 1 | 1 | 1 | 1 | 1 |
| Coloured | 0.77 (0.58 – 1.03) | 1.47 (1.09 – 1.97)^*^ | 0.55 (0.42 – 0.71)^*^ | 0.76 (0.46 – 1.23) | 1.16 (0.89 – 1.51) | 0.54 (0.44 – 0.66)^*^ |
| ***Level of education*** |  |  |  |  |  |  |
| No schooling | 1 | 1 | 1 | 1 | 1 | 1 |
| Primary | 1.50 (1.09 – 2.06)^*^ | 1.29 (0.87 – 1.93) | 1.25 (0.93 – 1.67) | 1.10 (0.66 – 1.83) | 1.04 (0.75 – 1.42) | 1.53 (1.20 – 1.95)^*^ |
| Secondary | 0.90 (0.64 – 1.27) | 1.45 (1.01 – 2.22)^*^ | 1.03 (0.75 – 1.39) | 0.83 (0.48 – 1.45) | 0.86 (0.61 – 1.19) | 1.54 (1.20 – 1.98)^*^ |
| Tertiary | 1.02 (0.51 – 2.08) | 0.93 (0.37 – 2.32) | 0.54 (0.26 – 1.11) | 0.26 (0.03 – 1.99) | 0.87 (0.44 – 1.74) | 1.28 (0.77 – 2.15) |
| ***Smoking habits*** |  |  |  |  |  |  |
| Non-smoker | 1 | 1 | 1 | 1 | 1 | 1 |
| Ex-smoker | 1.40 (1.04 – 1.89)^*^ | 0.98 (0.68 – 1.42) | 1.54 (1.16 – 1.99)^*^ | 2.00 (1.24 – 3.24)^*^ | 1.31 (1.04 – 1.82)^*^ | 0.93 (0.74 – 1.18) |
| Current smoke | 1.04 (0.77 – 1.42) | 0.97 (0.69 – 1.39) | 1.30 (1.01 – 1.71)^*^ | 1.57 (1.15 – 2.59)^*^ | 0.82 (0.59 – 1.12) | 1.31 (1.05 – 1.65)* |
| ***Occupational exposure history to dust/chemical fumes*** |  |  |  |  |  |  |
| No | 1 | 1 | 1 | 1 | 1 | 1 |
| Yes | 1.22 (0.94 – 1.57) | 1.43 (1.07 – 1.91)^*^ | 0.93 (0.73 – 1.18) | 1.02 (0.66 – 1.57) | 0.80 (0.61 – 1.04) | 0.94 (0.77 – 1.14) |
| ***Main residential heating/cooking fuel type*** |  |  |  |  |  |  |
| Electricity | 1 | 1 | 1 | 1 | 1 | 1 |
| Gas | 1.65 (0.97 – 2.79) | 1.69 (0.91 – 3.16) | 0.76 (0.45 – 1.29) | 0.71 (0.21 – 1.57) | 1.35 (0.79 – 2.29) | 0.57 (0.35 – 1.14) |
| Paraffin | 1.81 (0.79 – 4.18) | 1.19 (0.41 – 3.48) | 2.03 (1.13 – 4.78)^*^ | 1.19 (0.27 – 5.12) | 2.40 (1.11 – 5.17)^*^ | 0.86 (0.41 – 1.79) |
| Open fires | 1.06 (0.23 – 4.83) | 1.29 (0.61 – 4.79) | 0.94 (0.25 – 3.53) | - | 2.18 (0.67 – 7.13) | 0.14 (0.02 - 1.12) |

1:Reference category

^*^Significant at P < 0.05

^a-f:^ Models adjusted for sex, age, population group, smoking habits, occupational exposure history to dust/chemical fumes and main residential heating/cooking fuel type.
